# Immune Checkpoint Inhibitor-Induced Insidiously Progressive, Fatal Interstitial Lung Disease

**DOI:** 10.3390/jpm15030115

**Published:** 2025-03-15

**Authors:** Nobuhiro Kanaji, Naoki Watanabe, Takuya Inoue, Hitoshi Mizoguchi, Yuta Komori, Yasuhiro Ohara, Norimitsu Kadowaki

**Affiliations:** Department of Internal Medicine, Division of Hematology, Rheumatology and Respiratory Medicine, Faculty of Medicine, Kagawa University, 1750-1 Ikenobe, Miki-cho, Kita-gun, Kagawa 761-0793, Japan

**Keywords:** immune checkpoint inhibitor, interstitial lung disease, prognosis, lung cancer

## Abstract

**Background/Objectives:** Immune checkpoint inhibitors (ICIs) cause interstitial lung diseases (ILDs) as a type of immune-related adverse event (irAE). The characteristics of ICI-ILD are diverse. The objective of this study is to investigate the clinical features of ICI-ILD, with particular emphasis on insidiously progressive ICI-ILD. **Methods:** We retrospectively analyzed 232 patients with advanced lung cancer who were treated with ICIs (including combination therapy with cytotoxic agents). **Results:** IrAEs were observed in 85 patients (36.6%). The most frequent irAE was ICI-ILD (41 patients, 17.7% of all patients). The occurrence of ICI-ILD was associated with a significantly better response compared to the non-irAE group (response rates: 88% vs. 33%), longer progression-free survival (PFS) (median: 17.5 vs. 3.0 months), and longer overall survival (median: 52.6 vs. 16.6 months), respectively. However, six patients died from ICI-ILD, which could be divided into two patterns: early-onset ICI-ILD in three patients (median PFS: 1.2 months), and insidiously progressive ICI-ILD in three patients. In the latter type, ICI-ILD developed unnoticed, progressed insidiously, and led to respiratory failure (median PFS: 7.2 months). The non-organizing pneumonia pattern and a weak response to corticosteroid therapy were also common findings. On average, six cycles of ICI treatment were administered between the time when ICI-ILD became retrospectively recognizable and the discontinuation of ICI treatment. During this period, C-reactive protein levels and the extent of ILD involvement gradually increased. **Conclusions:** Insidiously progressive ICI-ILD can lead to fatal outcomes. Early discontinuation of ICIs upon recognition of this type of ICI-ILD may improve patient outcomes.

## 1. Personalized Medicine

Personalized medicine for patients with lung cancer has made remarkable progress. The cornerstone of personalized medicine includes molecular targeted therapies and immunotherapies. This study describes the characteristics of immune checkpoint inhibitor-related interstitial lung diseases (ICI-ILDs) for patients with advanced lung cancer and the characteristics of ICI-ILDs in which ICI should be discontinued early. In particular, we focus on “insidiously progressive ICI-ILD”, which can result in fatal outcomes.

## 2. Introduction

Personalized medicine for patients with lung cancer has made remarkable progress. The cornerstone of personalized medicine includes molecular targeted therapies and immunotherapies, which are tailored based on driver gene status and programmed cell death-ligand 1 (PD-L1) expression. Immune checkpoint inhibitors (ICIs) are antibodies that target immune checkpoint molecules, such as programmed cell death-1 (PD-1) and PD-L1, and exert antitumor effects by activating the host’s immune system. ICIs now play a central role in immunotherapy for lung cancer. Clinical trials targeting advanced lung cancer have shown that treatment with ICIs, both alone and in combination with cytotoxic agents, resulted in better clinical outcomes compared to treatment with cytotoxic agents alone [1,2]. However, excessive activation of the host immune system can lead to immune-related adverse events (irAEs), which may affect various organs. Among these irAEs, ICI-related interstitial lung diseases (ICI-ILDs), in particular, can result in fatal outcomes. In clinical trials, the frequency of ICI-ILD has been reported to be approximately 5% [1,2], while real-world data, especially from Japan, have shown a higher incidence, ranging from 12.4% to 18.6% [3,4,5,6].

In 144 reported cases of lung injury caused by nivolumab, radiographic patterns included organizing pneumonia (OP), hypersensitivity pneumonitis (HP), diffuse alveolar damage (DAD), nonspecific interstitial pneumonia (NSIP), etc., with OP being the most frequent [7]. The radiologic OP pattern is characterized by areas of consolidation, often in a predominantly peripheral or peribrochovascular distribution [8]. Several studies have reported that ICI-ILDs occurring within several months after the initiation of ICI treatment generally present with higher grades, including grade 5 ILDs [3,4]. However, ICI-ILDs can occur at any point during treatment and may sometimes progress insidiously over several months. When ICI-ILD develops rapidly, subjective symptoms such as fever, cough, or dyspnea may occur, and chest imaging, along with certain blood tests, such as C-reactive protein (CRP), may show abnormal findings. On the other hand, when ICI-ILD develops gradually, these symptoms and abnormalities may be minimal, and there are no reports on the usefulness of blood tests for detecting early ICI-ILD. Although the presence of subjective symptoms is associated with the severity of ICI-ILD [9], data on chronic progressive ICI-ILDs, particularly in relation to the speed of progression, remain limited. The present study aims to investigate the clinical features of ICI-ILDs, including “insidiously progressive ICI-ILD” in patients with advanced lung cancer.

## 3. Materials and Methods

### 3.1. Patients

Patients with pathologically confirmed advanced lung cancer who presented to the Department of Internal Medicine, Kagawa University Hospital, between July 2012 and December 2023 were retrospectively identified, and relevant clinical and laboratory data were collected from their medical records. Patients treated with ICIs in the first or later line were included in this study. Both ICI alone or in combination with cytotoxic anticancer drugs were included. However, patients with locally advanced disease who had undergone definitive thoracic irradiation and subsequent ICI consolidation therapy were excluded. Patients who had undergone surgery with curable intent or had another concomitant active malignancy were also excluded. Patients were not excluded based on preexisting ILD, driver gene status, or PD-L1 status.

### 3.2. Evaluation of ILDs

General blood tests, including CRP, lactate dehydrogenase (LDH), and white blood cell (WBC) count, as well as chest X-rays, were routinely performed at each visit for ICI treatment. Chest computed tomography (CT), spirometry, and blood tests, such as von den Lungen-6 (KL-6), were conducted when deemed necessary by the treating physician. The evaluation of ILDs on high-resolution CT was performed in accordance with the ATS/ERS/JRS/LATA statement [8,10]. ICI-ILDs were diagnosed based on the patients’ clinical features, radiologic findings, and bronchoscopic examination when available. In accordance with the sequential reading method, at least two board-certificated pulmonologists independently reviewed all of the patients’ CT scans and classified the ILD patterns. The toxicity severity of ICI-ILD was determined according to the Common Terminology Criteria for Adverse Events (CTCAE) (https://ctep.cancer.gov/protocoldevelopment/electronic_applications/ctc.htm (assessed on 8 March 2025)) and was briefly summarized in a recent review [11]. The treatments for ICI-ILDs were determined by the treating physician based on the clinical severity of ICI-ILD.

### 3.3. Statistical Analysis

Progression-free survival (PFS) was defined as the duration of time from the start of ICI treatment (an ICI, two ICIs, or combination therapy with an ICI and cytotoxic agents) to disease progression or death. Overall survival (OS) was defined as the duration of time from the diagnosis of lung cancer to death. PFS and OS curves were constructed by using the Kaplan–Meier method, and the differences in survival were assessed by the log-rank test. Multivariate analysis of PFS was performed with the Cox proportional hazards model. Fisher’s exact test was used to compare the frequency of ICI-ILD. Fisher’s exact test and Student’s *t*-test were used to analyze risk factors for ICI-ILD deaths. All statistical analyses were performed using BellCurve for Exel version 4.08 (Social Survey Research Information, Tokyo, Japan).

## 4. Results

### 4.1. Clinical Characteristics and ICI Responsiveness

Table 1 Characteristics associated with PFS are listed in Table 2. Multivariate analysis showed that poor performance status, low PD-L1 expression, and second-line or later ICI treatment were independently associated with shorter PFS.

### 4.2. Immune-Related Adverse Events and ICI Responsiveness

**Figure 1:**
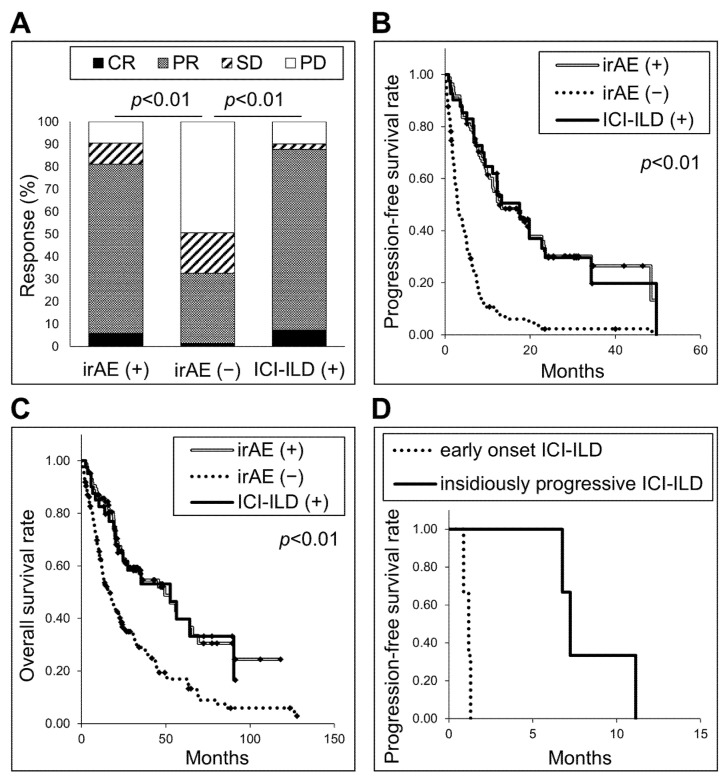
(**A**) The responses of the patients with or without immune-related adverse events (irAEs) or immune checkpoint inhibitor-related interstitial lung disease (ICI-ILD). *p* < 0.01 compared to irAE (−). (**B**) Kaplan–Meier curves of the progression-free survival (PFS) of the patients with or without irAEs or ICI-ILD. *p* < 0.01 vs. irAE (−). (**C**) Kaplan–Meier curves of the overall survival of the patients with or without irAEs or ICI-ILD. *p* < 0.01 vs. irAE (−). (**D**) Kaplan–Meier curves of PFS of patients who died of ICI-ILD.

IrAEs were observed in 85 of the 232 patients (36.6%). Among the irAEs, ICI-ILD was the most frequent, affecting 41 patients (17.7% of the 232 patients). The occurrence of irAEs or ICI-ILD was associated with a significantly better response rate compared to non-irAEs (response rates: 81% or 88% vs. 33%, respectively, *p* < 0.01, Figure 1A), significantly longer PFS (median PFS: 12.6 or 17.5 vs. 3.0 months, respectively, *p* < 0.01, Figure 1B), and significantly longer OS (median OS: 49.4 or 52.6 vs. 16.6 months, *p* < 0.01, Figure 1C). ICI-ILD was observed in two of 45 patients with driver gene mutations/fusions; one was the epidermal growth factor receptor (EGFR) mutation-positive patient shown later as patient 1. The other was a Kirsten rat sarcoma viral oncogene homolog (KRAS)-positive patient. The frequency of ICI-ILD was lower in patients with EGFR mutation compared with patients without driver gene mutations/fusions (3.3% vs. 20.9%, respectively, *p* = 0.02). Similarly, the frequency of ICI-ILD was lower in patients with driver gene mutations/fusions compared with those without (4.4% vs. 20.9%, respectively, *p* = 0.01).

### 4.3. Characteristics Associated with Death Due to ICI-ILD

Six patients died from ICI-ILD (15% of patients with ICI-ILD). Table 3 shows the characteristics associated with death from ICI-ILD. The characteristics of the ICI-ILDs that resulted in death included a non-organizing pneumonia (non-OP) pattern, involvement in all five lobes, higher grade (a median of 4), and elevated CRP levels at the time of ICI discontinuation. Retrospective analysis of the process leading up to ICI discontinuation revealed that the extent of ILD expanded, and CRP levels increased from the time when ILD became visible on CT scans. Further examination revealed that the PFS of patients who died from ICI-ILD could be divided into two patterns (Figure 1D). Three patients died from early-onset ICI-ILD, while in the other three patients, the ICI-ILD developed unnoticed, progressed insidiously, and ultimately led to death due to respiratory failure (PFS: 6.8, 7.2, and 11.1 months). We refer to the ICI-ILD observed in these three patients as “insidiously progressive ICI-ILD”.

### 4.4. The Three Patients Who Died of Insidiously Progressive ICI-ILD

**Figure 2:**
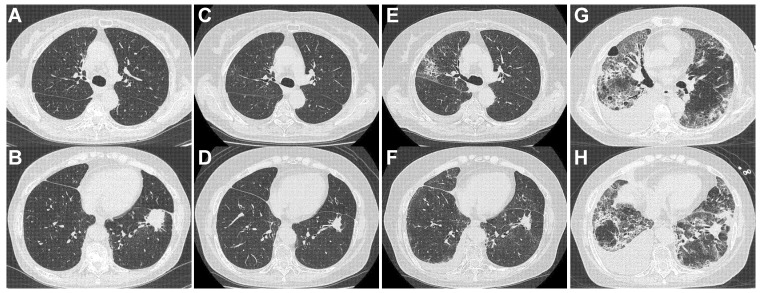
Case 1, a 65-year-old female. CT images. (**A**,**B**) Before pembrolizumab. (**C**,**D**) After 13 cycles of pembrolizumab. (**E**,**F**) After 22 cycles of pembrolizumab. (**G**,**H**) After an additional 3 months of corticosteroid therapy.

**Figure 3:**
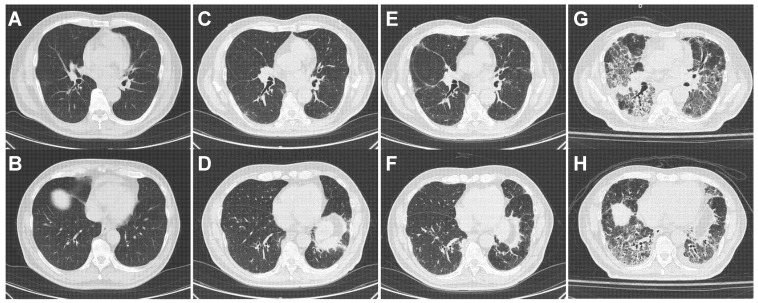
Case 2, a 68-year-old man. CT images. (**A**,**B**) Before treatment. (**C**,**D**) After four cycles of carboplatin, pemetrexed, and pembrolizumab. (**E**,**F**) After an additional four cycles of pembrolizumab. (**G**,**H**) After an additional 3 months with best supportive care.

**Figure 4:**
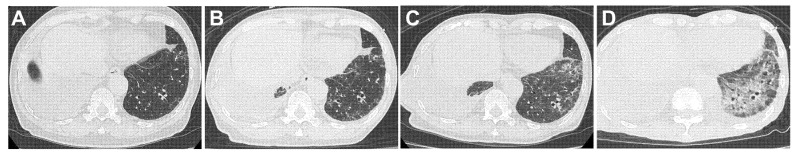
Case 3, an 81-year-old man. CT images. (**A**) Before pembrolizumab. (**B**) After 11 cycles of pembrolizumab. (**C**) After 17 cycles of pembrolizumab. (**D**) After an additional 5 months with best supportive care.

Table 4 shows the characteristics of patients who died from insidiously progressive ICI-ILD, and Figure 2, Figure 3 and Figure 4 show CT findings throughout the entire treatment course. In all three patients, the initial occurrence of ICI-ILD was localized and subtle, such that the physicians did not notice it. As a result, ICI treatment was continued, and ILD progressed insidiously before treatment discontinuation. After discontinuation of ICI treatment, ILD improvement was poor despite corticosteroid therapy, and the patients eventually died from respiratory failure. An average of six cycles of ICI treatment were administered between the development of ICI-ILD and the discontinuation of ICI treatment. During this period, CRP, LDH, and WBC counts in peripheral blood, the extent of ILD involvement, and ILD grade gradually increased.

#### 4.4.1. Patient 1 (Figure 2, Table 4)

A 65-year-old Japanese woman was treated with erlotinib as second-line therapy. A 45-day interval was allowed after the completion of erlotinib, and then pembrolizumab was initiated as third-line therapy. After 22 cycles of pembrolizumab, the patient’s physician noted the development of ICI-ILD (grade 2) on a CT scan performed to assess lung cancer, and pembrolizumab was discontinued (Figure 2E,F). At this time, the patient had a mild cough as the only symptom. Retrospective observation revealed that ICI-ILD had already developed after 13 cycles of pembrolizumab (Figure 2C,D, grade 1). Although corticosteroid treatment was initiated (prednisolone 0.5 mg/kg), after the discontinuation of pembrolizumab, the patient’s ICI-ILD progressed (Figure 2G,H), and she died from respiratory failure without responding to methylprednisolone (mPSL) pulse therapy.

#### 4.4.2. Patient 2 (Figure 3, Table 4)

Carboplatin, pemetrexed, and pembrolizumab were administered as the first-line therapy to this 68-year-old Japanese man. After four cycles of this combination therapy, the patient exhibited a partial response. Following four additional cycles of pembrolizumab maintenance therapy, the patient’s physician noticed the occurrence of ICI-ILD (Figure 3E,F, grade 1) on a CT scan performed to assess lung cancer, and pembrolizumab was discontinued. At that time, the patient had no symptoms. Retrospective observation revealed that ICI-ILD had already developed after four cycles of first-line combination therapy (Figure 3C,D). After an additional three months of best supportive care, the ICI-ILD worsened (Figure 3G,H, grade 4), and the patient eventually died from respiratory failure despite the administration of corticosteroids, including mPSL pulse therapy.

#### 4.4.3. Patient 3 (Figure 4, Table 4)

Pembrolizumab monotherapy was administered as first-line therapy for this 81-year-old Japanese man. After 17 cycles of pembrolizumab, the patient’s physician observed the occurrence of ICI-ILD (Figure 4C, grade 2) on CT scans performed to evaluate lung cancer, and the pembrolizumab was discontinued. The patient had no symptoms at that time. Retrospective analysis revealed that ICI-ILD had already developed after 11 cycles of pembrolizumab (Figure 4B, grade 1). Although corticosteroid therapy was initiated after pembrolizumab was discontinued, ICI-ILD progressed five months later (Figure 4D, grade 4), and he eventually died from respiratory failure.

## 5. Discussion

This retrospective analysis of 232 patients treated with ICIs for advanced lung cancer demonstrated that the occurrence of ICI-ILD was associated with a high response rate (88%) and better survival outcomes (median [m]PFS 17.5 months and mOS 52.6 months). However, some cases of ICI-ILD progressed insidiously and showed a weak response to corticosteroid therapy, leading to fatal outcomes.

The occurrence of irAEs is well known to be associated with better outcomes [12,13,14,15]. A survival analysis adjusted for relevant clinical factors demonstrated that even after accounting for immortal time bias, pembrolizumab and chemotherapy-induced pneumonitis were independently associated with shorter survival [3]. However, in the present population, the survival of patients with ICI-ILD (including six patients who died from ICI-ILD) was significantly longer than that of patients without irAEs. This finding clearly suggests that ICI-ILDs do not always result in poor outcomes. Consistent with this, some reports documented that ICI-ILD correlates with ICI efficacy [16,17]. In a report of 39 patients who experienced irAE, response rate and PFS were significantly better in patients with ICI-ILD than in patients with irAEs-non-ILD and non-irAEs [16]. In another report of 22 patients with ICI-ILD, OS was significantly shorter in patients with severe grade ICI-ILD than in those without severe grade (*p* = 0.011; median OS 3.0 vs. 12.7 months, respectively) [17]. The pooled analyses of phase 3 randomized clinical trials using atezolizumab showed that even grade 3 to 5 irAEs are not a poor prognostic factor if the acute phase can be overcome [18]. We believe that manageable ICI-ILDs can be considered favorable events, similar to most other irAEs.

Several studies have shown that fatal ICI-ILDs occur in the early phase after the initiation of ICI treatment. In a retrospective study of patients treated with a combination of platinum, pemetrexed, and pembrolizumab [3], all 10 patients with grade 3 or higher ICI-ILD developed within 4 months of starting this combination therapy. In a prospective study of 138 patients who received anti-PD-1 monotherapy [4], all eight patients with grade 3 or worse ICI-ILD (including three patients with grade 5 ILD) developed ICI-ILD within 60 days of initiating the ICI monotherapy. Consistent with these reports, in the present study, three of the six patients who died from ICI-ILD experienced early-onset ILD after the initiation of ICI therapy, which progressed acutely. However, in the remaining three patients, ICI-ILD progressed insidiously and ultimately led to fatal outcomes. This finding represents the most important contribution of our study.

Three patients in the present series died from insidiously progressive ICI-ILD that developed more than three months after the initiation of ICI therapy. In these three fatal cases, the behavior of ICI-ILD was notably similar: insidious progression, a non-OP pattern (initially presenting as an NSIP or unclassified IIP pattern and eventually evolving into a DAD pattern), and a weak response to corticosteroid therapy. In contrast, all patients with the OP pattern recovered from ICI-ILD. The OP pattern, characterized by consolidation, is relatively easy to detect on a chest X-ray or CT scan, whereas the non-OP pattern, which lacks significant changes on X-rays, is more difficult to identify. This raises the critical question of how clinicians can best recognize the development of insidiously progressive ICI-ILD. In this contest, peripheral blood tests might serve as valuable tools for the early detection of ICI-ILD. However, to our knowledge, there are no reports of blood tests being helpful for detecting early ICI-ILD, although several studies reported that KL-6, interleukin-9, and other factors before initiation of ICI are useful in predicting the development of ICI-ILD [19,20]. In the present patient series, classical ILD markers such as CRP, LDH, and WBC levels gradually increased during the progression of ICI-ILD. Since the early non-OP pattern is often difficult to detect using plain chest X-rays alone, we recommend close monitoring of classical ILD markers. In patients with abnormal blood test results, a chest CT examination should be performed promptly to avoid delays in ICI discontinuation. Among patients with ICI-ILD, those with subjective symptoms exhibited a higher grade (3–5) of ICI-ILD than those whose ICI-ILD was detected during routine examinations [9]. However, deaths have also occurred among patients whose ICI-ILD was discovered through routine examinations. Pulmonary function tests, KL-6, and autoantibody screening were not routinely conducted in this study. Therefore, the utility of these tests or markers for the early detection of insidiously progressive ICI-ILD remains unclear.

It remains unknown whether early treatment interventions for insidiously progressive ICI-ILD can prevent fatal outcomes. Corticosteroids did not demonstrate sufficient efficacy to prevent the death of the three patients described above. In the INBUILD trial, it was reported that among patients with progressive fibrosing ILDs, the annual rate of decline in forced vital capacity was significantly lower in patients who received nintedanib compared to those who received a placebo [21]. Anti-fibrotic agents such as nintedanib might also be effective in preventing the progression of insidiously progressive ICI-ILD.

Several clinical studies have demonstrated the limited efficacy of ICI monotherapy in patients with EGFR mutations. Although OS was longer in patients treated with ICI monotherapy than in those treated with docetaxel, no OS benefit of ICI monotherapy was observed in subgroup analysis of patients with EGFR mutations in the CheckMate 057 and KEYNOTE-010 studies [22,23]. In the ATLANTIC study, 111 patients in cohort 1 had EGFR mutation-positive or ALK fusion-positive tumors [24]. Among patients with PD-L1 expression greater than 25%, an objective response was achieved in 9 (12.2%) of 74 patients. In fact, some EGFR-positive patients respond dramatically, even to ICI monotherapy [25,26]. The duration of response to prior EGFR-TKIs may predict ICI responsiveness in patients with EGFR mutations [27]. In a subgroup analysis of patients with EGFR mutations of the IMpower 150 trial, improved OS was observed in patients treated with atezolizumab plus bevacizumab plus carboplatin plus paclitaxel (ABCP) compared with those without atezolizumab [28]. In the phase III ATTLAS trial, 228 patients with EGFR mutation (n = 215) or ALK fusion (n = 13) were enrolled [29]. Objective response rates (69.5% vs. 41.9%, *p* < 0.01) and PFS (median 8.48 vs. 5.62 months, hazard ratio 0.62, *p* = 0.004) were significantly better in the ABCP group than in the pemetrexed plus carboplatin or cisplatin group [29]. Based on these results, ABCP, rather than ICI monotherapy, can be recommended for patients with EGFR mutations who tolerate chemotherapy. Regarding the frequency of ICI-ILD in patients with EGFR mutations, there were two treatment-related deaths (1.3%) due to ILD in the ABCP arm of the ATTLAS trial [29]. In the present study, the frequency of ICI-ILD was lower in patients with EGFR mutations compared with those without driver gene mutations/fusions (3.3% vs. 20.9%, respectively). Thus, regardless of EGFR mutation status, the frequency of ILD correlates with the efficacy of ICIs. In the present study, one patient who died of ICI-ILD was positive for EGFR mutation (patient 1). The patient received second-line erlotinib and third-line pembrolizumab, with a 45-day interval between the two drugs. The blood half-life of erlotinib has been reported to be short, approximately 26 h [30]. After a 45-day interval, erlotinib in the body is expected to be virtually eliminated. ILD due to EGFR-TKI occurs during oral administration, including cases of re-administration [31], and the development of ILD after TKI discontinuation is not common. In the analysis of 353 patients using a claims database, pre-treatment with EGFR-TKI was not associated with ICI-ILD [32]. Based on these findings, we think it is unlikely that pre-treatment with erlotinib had any effect on the development of ILD after pembrolizumab in patient 1.

The limitations of this study are as follows. First, it was a retrospective, single-center investigation. The patient management strategy during ICI treatment, including the intervals of CT scans and the interpretation system for radiological examinations, varies among facilities. Such differences could influence the early diagnostic accuracy of ICI-ILD, the treatment approach, and patient outcomes. Second, the diagnosis of ICI-ILD in the present study was primarily based on patients’ clinical courses and radiological findings. Although bronchoscopic examinations were performed for some patients to rule out other conditions, such as infection, not all cases underwent such evaluations. Lastly, this study included both ICI therapy and combination therapy with ICI and cytotoxic agents. While some cases of treatment-related ILD might have been induced by cytotoxic agents rather than ICIs, the frequency of ILD caused by cytotoxic chemotherapy is known to be significantly lower than that of ICI-ILD [4,33,34].

## 6. Conclusions

ICI-ILD is generally associated with favorable response rates, PFS, and OS. However, it should be noted that insidiously progressive ICI-ILD exists and can lead to fatal outcomes. Pulmonologists should be aware of the occurrence of ICI-ILD presenting with a non-OP pattern. Early discontinuation of ICI at the onset of insidiously progressive ICI-ILD may improve patient outcomes.

## Figures and Tables

**Table 1:** Patient and tumor characteristics.

Characteristic	Patients (n = 232)
Age, median (range)	70 (41–94)
Male/female	182/50
Smoking history	
Smoker/never smoker	195/37
Pack-years in smokers, average (range)	54.5 (0.75–171)
ECOG Performance Status	
0/1/2/3/4	67/109/35/17/4
Histology	
Adenocarcinoma	132
Squamous cell carcinoma	45
Non-small cell carcinoma, NOS	12
Others	16
Small cell carcinoma	27
Driver mutations	
*EGFR*/*ALK*/others/no mutations	30/3/12/84
Clinical stage (8th edition of TNM classification)	
III/IV	27/205
PD-L1 TPS (%)	
<1/1–49/50≤/not evaluated	42/55/71/64
ICI Regimens	
ICI monotherapy	
nivolumab	39
pembrolizumab	51
atezolizumab	18
ICI + chemotherapy	
pembrolizumab + carboplatin + PEM	28
pembrolizumab + carboplatin + nab-PAC	22
atezolizumab + carboplatin + etoposide	16
atezolizumab + carboplatin + PEM	12
atezolizumab + carboplatin + nab-PAC	11
durvalumab + carboplatin + etoposide	10
ipilimumab + nivolumab	9
ipilimumab + nivolumab + carboplatin + PEM	6
tremelimumab + durvalumab + carboplatin + nab-PAC	6
others	4
Therapeutic line of ICI	
1/2/3/4/5 or later	148/47/16/10/11

*ALK*: anaplastic lymphoma kinase, ECOG: Eastern Cooperative Oncology Group, *EGFR*: epidermal growth factor receptor, NOS: not otherwise specified, TPS: tumor proportion score, ICI: immune checkpoint inhibitor, PEM: pemetrexed, and PAC: paclitaxel.

**Table 2:** Characteristics associated with PFS.

Characteristic	n	Median PFS, Days	Univariate Analysis *p*-Value	Multivariate Analysis	
				HR (95%CI)	*p*-Value
Age					
Older, ≥75	57	202	0.69		
Younger, <75	135	154			
Gender					
Male	151	172	0.56		
Female	41	140			
Smoking history					
Never smoker	32	83	0.17		
Smoker	160	181			
ECOG Performance Status					
2−4	66	74	<0.01	2.22 (1.45–3.38)	<0.01
0−1	126	217			
Histology					
SCLC	23	183	0.50		
NSCLC	169	168			
Driver mutations					
Yes	32	140	0.81		
No	160	172			
PD-L1 TPS					
<50	80	171	<0.01	1.68 (1.17–2.42)	<0.01
50≤	51	241			
Preexisting ILD					
Yes	26	126	0.19		
No	166	172			
Therapeutic line of ICI					
2nd or later	79	68	<0.01	2.51 (1.66–3.81)	<0.01
1st	113	225			
Therapeutic regimen					
IO only (one or two ICIs)	103	88	0.12		
IO plus chemotherapy	89	203			
CRP (mg/dL)					
1≤	106	154	0.16		
<1	85	183			
LDH (U/L)					
220≤	77	119	0.24		
<220	28	191			
NLR					
5≤	72	94	<0.01	0.65 (0.42–1.00)	0.05
<5	119	203			
PNI					
<40	79	119	<0.01	0.82 (0.53–1.28)	0.38
40≤	111	205			

**Table 3:** Characteristics associated with ICI-ILD death.

Characteristic	Survival (n = 35)	Death (n = 6)	Univariate Analysis *p*-Value
At ICI discontinuation			
ICI-ILD pattern, non-OP/OP (n)	18/17	6/0	0.03
ICI cycle number	7 ± 6	9 ± 8	0.80
CRP (mg/dL)	4.6 ± 5.0	10.4 ± 5.0	<0.05
LDH (U/L)	299 ± 214	394 ± 151	0.25
WBC (/microL)	7623 ± 2935	9772 ± 3402	0.23
Number of lung lobes affected by ICI-ILD	4 ± 1	5 ± 0	<0.01
ICI-ILD grade	2 ± 1	4 ± 1	<0.01
Between ICI initiation and ICI-ILD development			
Period (days)	159 ± 133	106 ± 104	0.34
Between ICI-ILD development and ICI discontinuation			
Period (days)	31 ± 67	89 ± 92	0.23
Additional ICI cycle number	1 ± 3	3 ± 3	0.27
Change in CRP	0.01 ± 1.06	4.51 ± 3.78	0.04
Change in LDH	3 ± 27	127 ± 160	0.14
Change in WBC	627 ± 2055	2588 ± 2432	0.14
Change in lung lobes affected by ICI-ILD	0 ± 1	2 ± 2	0.03
Change in ICI-ILD grade	0 ± 1	2 ± 1	0.08

**Table 4:** Three patients died of insidiously progressive ICI-ILD.

Patient No.	Patient 1	Patient 2	Patient 3
Age	65	68	81
Gender	F	M	M
Smoking (pack-year)	0	30	48
Histology	adenocarcinoma	adenocarcinoma	adenocarcinoma
Clinical stage	cT1bN0M1b (OSS), IVA	cT3N2M1c (ADR, LYM),	cT3N0M1a (PLE), IVA
		IVB	
1st line therapy	carboplatin + pemetrexed	carboplatin + pemetrexed+	pembrolizumab
		pembrolizumab	
2nd line therapy	erlotinib		
3rd line therapy	pembrolizumab		
Days between ILD	197	119	211
Development and ICI			
discontinuation			
At ICI-ILD development and at ICI discontinuation			
ICI cycle number	13 and 22	4 and 8	11 and 17
CRP (mg/dL)	0.13 and 2.92	0.15 and 8.49	1.1 and 7.76
LDH (U/L)	188 and 215	210 and 543	214 and 586
WBC (/microL)	4510 and 7180	4900 and 10,850	4300 and 9910
Lobes of ICI-ILD	2 and 5	2 and 5	2 and 5
ICI-ILD grade	1 and 2	1 and 4	1 and 4

CRP: C-reactive protein, ICI: immune checkpoint inhibitor, ILD: interstitial lung disease, LDH: lactate dehydrogenase.

## Data Availability

The datasets generated and analyzed during the current study are not publicly available due the publication of raw data have not been approved by the Research Ethics Committee of Kagawa University (No. 2024-043).

## Abbreviations

The following abbreviations are used in this manuscript.

CRP	C-reactive protein
DAD	diffuse alveolar damage
EGFR	epidermal growth factor receptor
HP	hypersensitivity pneumonitis
ICI	immune checkpoint inhibitor
ILD	interstitial lung disease
irAE	immune-related adverse event
KL-6	Krebs von den Lungen-6
LDH	lactate dehydrogenase
